# CD38: From Positive to Negative Expression after Daratumumab Treatment

**DOI:** 10.7759/cureus.7627

**Published:** 2020-04-10

**Authors:** Bonell Patiño-Escobar, Roberto Ramos, Maximo Linares, Angie Mejía, Sebastián Alcalá

**Affiliations:** 1 Hematology, Instituto Nacional de Cancerología, Bogota, COL

**Keywords:** multiple myeloma, relapsed, refractory

## Abstract

CD38 is a glycoprotein expressed at a low level in myeloid and lymphoid tissues. However, it is highly and homogeneously expressed in plasma cells (PC) in multiple myeloma. Daratumumab is a human CD38-specific IgG1 antibody available for the treatment of multiple myeloma in Colombia. It has been authorized in relapsed/refractory disease as front-line treatment for non-eligible stem cell transplantation patients by INVIMA (Instituto Nacional de Vigilancia de Medicamentos y Alimentos) that is the regulatory agency. Daratumumab treatment has been associated with the negativization of the expression of CD38 in PC, demonstrating a resistance mechanism under the clonal evolution theory. We report the case of a 63-year-old male, diagnosed with a relapsed/refractory multiple myeloma, heavily treated, who expressed strong CD38 marker at the beginning of the treatment, with a posterior negativization of CD38 after four cycles of treatment with daratumumab.

## Introduction

Multiple myeloma is a hematopoietic disease in which the clonal expansion of neoplastic plasma cells (PC) disrupts bone marrow microenvironment, related to a para-protein secreted (clonal gamma globulin), which ends up causing tissue damage. During the disease duration, different treatment types are used in an attempt to eradicate the clonal cells to ultimately cure the multiple myeloma. In the last decade, several monoclonal antibodies have been developed against specific targets within tumor cells, as the CD38 antibodies like daratumumab [1-3].

CD38 is a transmembrane glycoprotein type II which improves migration, adhesion cellular signaling over normal conditions in PC. Nevertheless, in multiple myeloma, an overexpression becomes a specific target to treat the disease. Daratumumab joins to the tail of the Fc portion of FC gamma receptors (FcyR), which are in the effector immune cells. It allows the induction of PC death through three mechanisms: complement-dependent cytotoxicity, antibody-dependent cytotoxicity, and antibody-dependent cellular phagocytosis. Thus, the union of FcyR ultimately induces programmed death. The negativization of CD38 expression in PC is supposedly a resistant mechanism under clonal evolution. Also, different mechanisms of primary and acquired resistance related to CD38 expression have been documented [4,5].

The mechanism of daratumumab-induced resistance is not completely clear. However, multiple in vitro researches have demonstrated some potential causes of daratumumab resistance. It is important to have in mind how much CD38 expression exists given the pivotal trials showed a greater possibility to reach response with CD38 antibodies within patients with higher expression of CD38 in PC compared to those who have lesser, as a starting point for describing the origin of the resistance. On the other hand, the downregulation of CD38 expression may be the main cause of acquired resistance against previous highly treated patients.

In November 2015, FDA approved daratumumab for the treatment of relapsed multiple myeloma [6]. However, in 2019 its use was introduced as the front-line treatment in patients eligible to transplantation combined with lenalidomide (MAIA trial) [7]. In Colombia, INVIMA (Instituto Nacional de Vigilancia de Medicamentos y Alimentos) approved daratumumab for relapsed/refractory multiple myeloma after two lines of treatment which, previously, included a proteasome inhibitor and immunomodulatory imide drugs (ImiDs) (POLLUX and CASTOR trials) [8,9]. Nevertheless, it has been approved recently as the front-line treatment for non-transplantation eligible patients based on the results of a clinical trial (ALCYONE trial) [10]. This work has been published as an abstract (https://www.clinical-lymphoma-myeloma-leukemia.com/article/S2152-2650(18)30885-1/abstract).

## Case presentation

A 63-year-old male patient diagnosed with IgA kappa multiple myeloma in 2011 treated with PAD regimen (Adriamycin, bortezomib, and dexamethasone) as front-line treatment. A complete response was reached and posterior consolidation with autologous stem cell transplantation was performed. He suffered relapse after two years. Several rescue treatments were given without any response: lenalidomide + dexamethasone, CyBorD, carfilzomib + dexamethasone, cyclophosphamide + dexamethasone, VRD (bortezomib + lenalidomide + dexamethasone).

**Figure 1:**
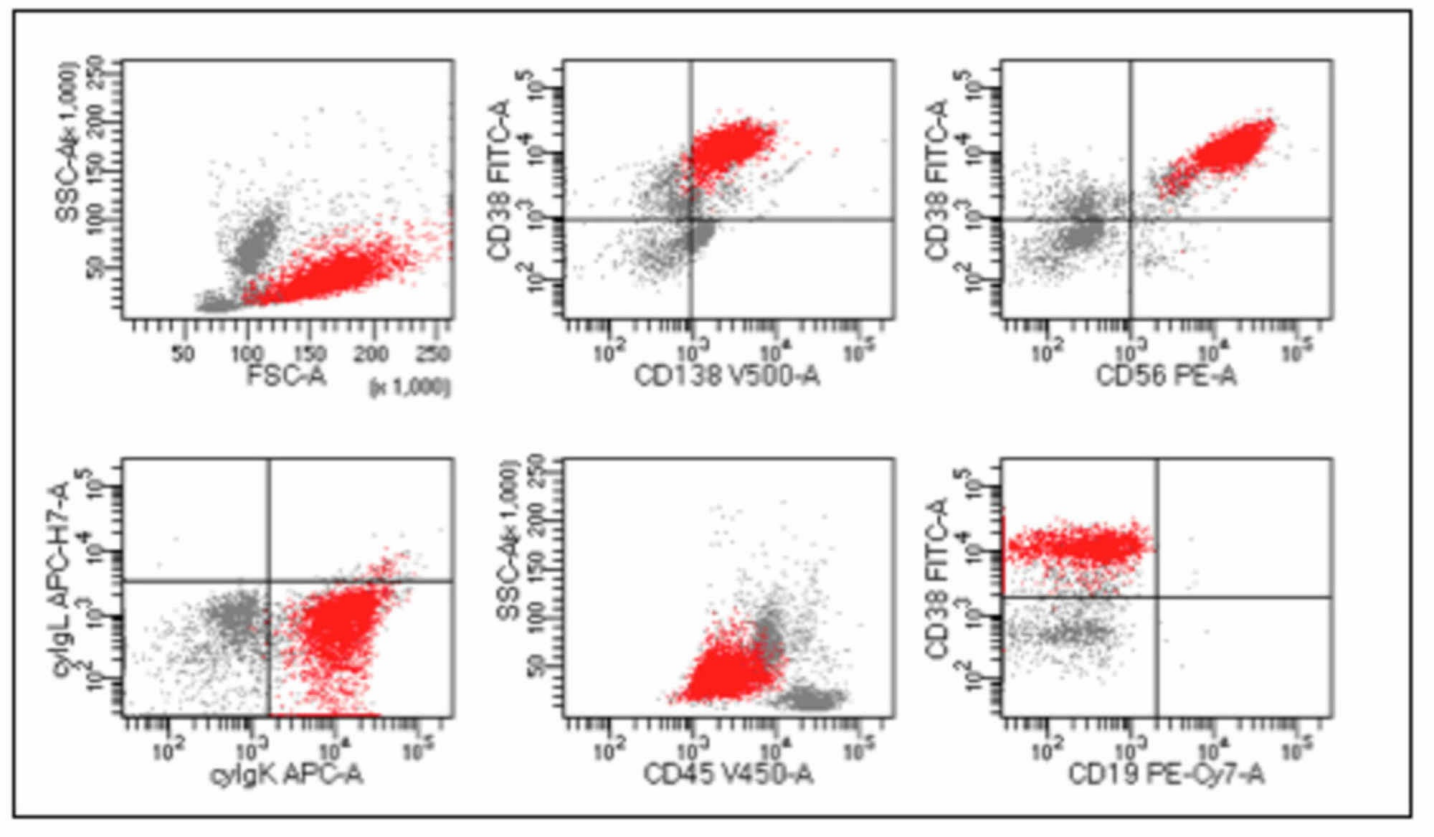
Flow cytometry showed 63.5% of myeloma cells (red events).

Just before the last relapse, flow cytometry showed 63.5% of PC with an expression of CD38+ (CD38/CD138: 63.5% and CD38/CD56: 63.5%), CD138, CD56, beta-2 microglobulin, and cytoplasmic kappa light chain, CD19-, CD45- (Figure 1). Afterward, a daratumumab + lenalidomide + dexamethasone regimen was started.

**Figure 2:**
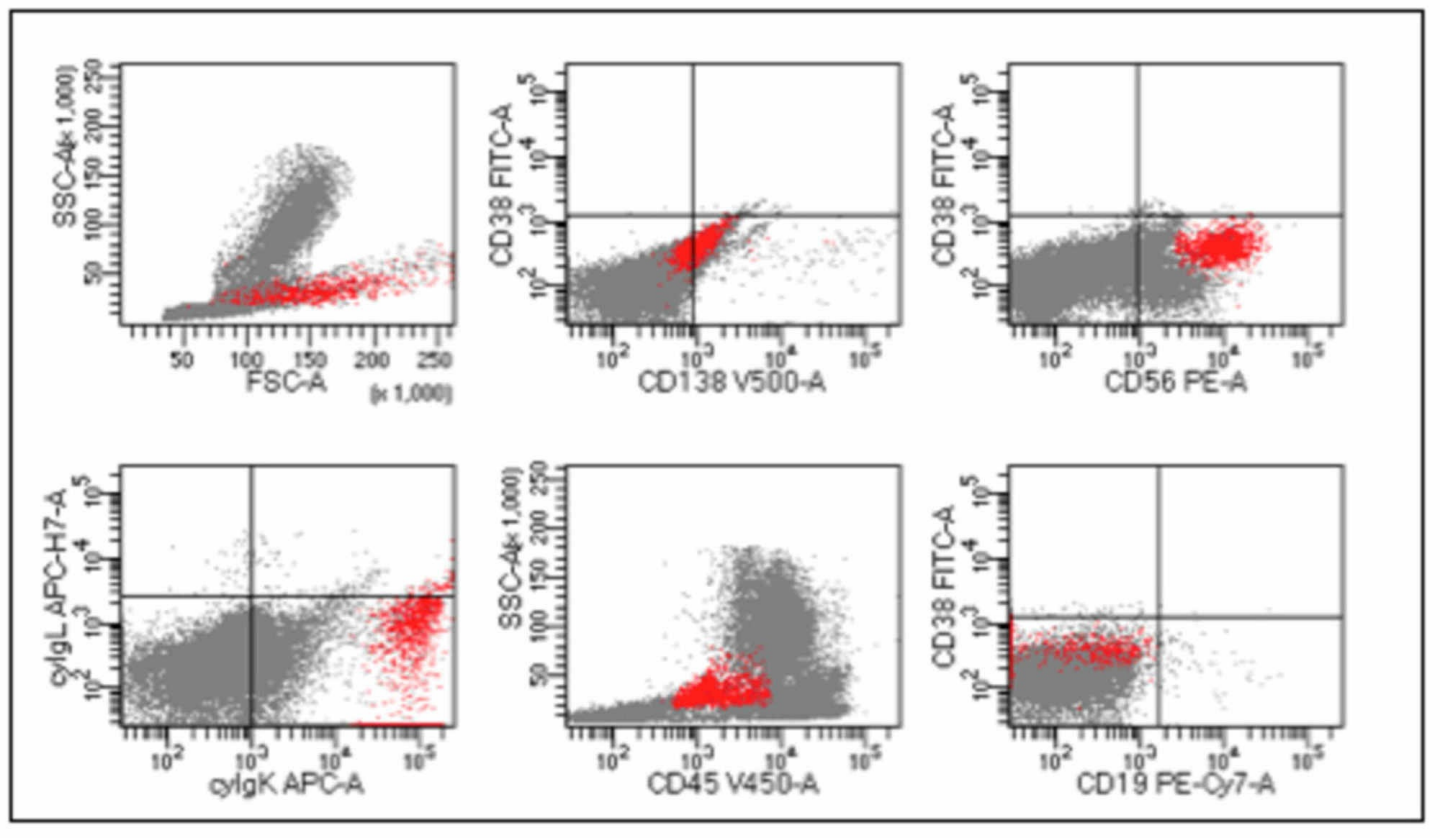
Flow cytometry showed 2.9% of myeloma cells (red events), CD38 negative.

After four cycles of treatment, a response evaluation was performed documenting partial response. Nevertheless, flow cytometry documented 4.8% of abnormal PC in bone marrow with an expression of CD38- (CD38-/CD138+: 4.8%, CD38-/CD56+: 4.8%), CD138+, CD56+, beta-2 microglobulin, cytoplasmic kappa light chain, CD19-, CD45- (Figure 2).

Due to the sustained clinical and biochemical response, daratumumab was continued. However, the patient suffered severe pneumonia requiring management in the intensive care unit. Accordingly, daratumumab was interrupted. Pomalidomide + cyclophosphamide + dexamethasone was ordered but it was not authorized by the health insurance.

**Figure 3:**
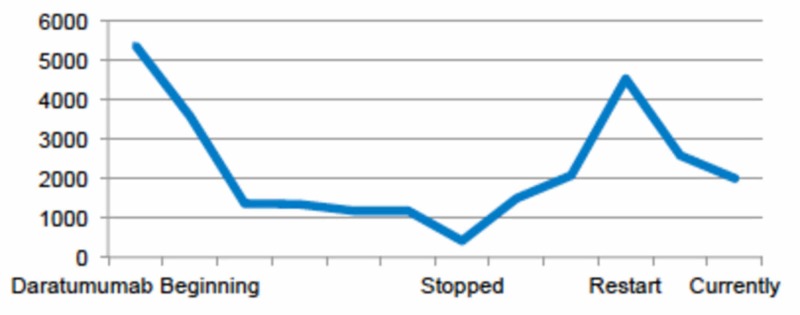
IgA plasma levels through treatment with daratumumab expressed in mg/dL.

Six months after daratumumab was ceased, the patient presented progression with M-protein rising and new bone lytic lesions. After multidisciplinary consideration, daratumumab was restarted with the same intensity of cycle 1 according to the POLLUX trial, plus antibiotic prophylaxis. CD38 was detected weak to negative expression by flow cytometry on bone marrow aspirate. Clinical improvement was documented early and the IgA levels reduced by 56% after just one cycle of re-challenge (Figure 3).

## Discussion

Daratumumab activates cytotoxic immune effector, antibodies-dependent cytotoxicity, antibodies-mediated phagocytosis and complement-dependent cytotoxicity. Furthermore, it modulates CD38 enzyme reducing adenosine levels (which normally produces an immunosuppressor activity), enhancing the immune response against tumor [1,3,5].

CD38 expression in PC is related to response to daratumumab treatment. Hence, we can divide the daratumumab into two categories: Primary and acquired resistance. Similar to other neoplasm treatments with monoclonal antibodies like Rituximab, Alemtuzumab, and Trastuzumab, the response depends on target antigen expression [11-14].

Treatment with daratumumab ends up also in a downregulation in myeloid-derived suppressor cells (MDSCs), regulatory T cell (Treg) and regulatory B cells (Bregs) activity. Furthermore, daratumumab produces and CD41 T-helper cells and CD81 cytotoxic T cells expansion. The measurement of IFN-Gamma levels indicates that due to T-cell clonality antiviral responses are increased after daratumumab treatment. These features indicate an appropriate T-cell response, despite low CD38 expression and may confirm T-cell response is due to depletion of regulatory cells. Those physiological changes are strong in patients who have shown any response to daratumumab, compared with those who did not. However, patients who have experienced relapse, showed loss of those features [5,15].

Complement-dependent cytotoxicity is one of the most important mechanisms of action against PC with daratumumab. It has been demonstrated reduction of CD55 and CD59 expression enhances daratumumab-mediated complement-dependent cytotoxicity in PC with high expression of CD38. In agreement, the expression of complement inhibitory proteins was not related to a response with daratumumab treatment, at least in monotherapy. Furthermore, CD55 and CD59 levels were higher in patients at the moment of progression compared with previous levels. A similar situation occurs in another neoplasm like chronic lymphocytic leukemia and rituximab, with this mechanism being a way of resistant for monoclonal antibodies due to impairment of complement-dependent cytotoxicity [1,16].

CD38 expression was detected weak to negative with our patient. This downregulation by internalization of CD38 after daratumumab exposure may explain a way of resistance. Nevertheless, several mechanisms could be feasible in the response shown in this case. Firstly, daratumumab will eliminate PC with higher CD38 levels of expression. Downregulation of CD38 might be a way to acquiring resistance to evade the immune response triggered by daratumumab, generally by internalization. The downregulation is temporary because, after six months of daratumumab cessation, CD38 expression rose up in PC. It has been demonstrated in other research, as it occurs with other monoclonal antibodies like rituximab in hematological diseases [1,2,4,17,18]. In the case described above, after six months of cessation of treatment with daratumumab, partial response was achieved, with clear evidence of increasing expression of CD38 in PC and possible re-sensitization of PC to daratumumab.

Nevertheless, reduction of CD38 expression after daratumumab exposure is possible in both, patients with refractory diseases and those who respond to daratumumab. Maintenance of low expression of CD38 might be a way of resistance by PC; however, patients who have demonstrated a response with daratumumab despite lack of CD38 expression may have biologic plausibility due to physiologic ligands for CD38 and CD31 expressed in matrix component. PC with a higher CD38 expression tend to have more adherence to stromal cells of bone marrow probably via CD38-CD31 interaction. Decreasing PC adhesion to bone marrow accessory cells via blockage of CD31-38 interaction might be a new potential mechanism to reach anti-multiple myeloma activity as well [4,5,19].

Even though CD38 reduction is common after daratumumab treatment, and it is related to the possibility of response, the upregulation of CD38 molecules enhances the response option with daratumumab in resistant PC clones as we have demonstrated in this case report. Thus, other approach for patients to become PC sensitive to daratumumab is to treat them with all-trans retinoic acid (ATRA). ATRA increases CD38 expression of PC, restoring expression of this molecule, but also reduces CD55 and CD59 levels improving astonishingly the complement-dependent cytotoxicity as one of the most important mechanisms of action of daratumumab [1,4].

## Conclusions

The negativization of CD38 expression in PC might be an evidence of resistant mechanism under clonal evolution. However, different mechanisms of loss of expression have been described: primary (lack of expression) and acquired (downregulation expression or hide receptor due to monoclonal antibodies). Regarding the resistance, the mechanism is not completely clear, but several in vitro researches have demonstrated some potential causes of daratumumab resistance and re-sensitization. This is an interesting pathway of further investigation in multiple myeloma: the search for re-sensitization of PC to the treatments currently available, not just ATRA for daratumumab, but other drugs for multiple myeloma like IMiDs, and proteasome inhibitors, which are highly effective.
